# Hydrogen Sulfide as a Potential Therapy for Heart Failure—Past, Present, and Future

**DOI:** 10.3390/antiox10030485

**Published:** 2021-03-19

**Authors:** Kyle B. LaPenna, David J. Polhemus, Jake E. Doiron, Hunter A. Hidalgo, Zhen Li, David J. Lefer

**Affiliations:** 1 Cardiovascular Center of Excellence, Louisiana State University Health Sciences Center, New Orleans, LA 70112, USA; klapen@lsuhsc.edu (K.B.L.); dpolhe@lsuhsc.edu (D.J.P.); jdoir4@lsuhsc.edu (J.E.D.); hhida1@lsuhsc.edu (H.A.H.); zli2@lsuhsc.edu (Z.L.); 2 Department of Pharmacology and Experimental Therapeutics, Louisiana State University Health Sciences Center, New Orleans, LA 70112, USA

**Keywords:** HFpEF, HFrEF, metabolic syndrome, obesity, cardiometabolic HFpEF, hydrogen sulfide therapeutics, hydrogen sulfide, heart failure

## Abstract

Hydrogen sulfide (H_2_S) is an endogenous, gaseous signaling molecule that plays a critical role in cardiac and vascular biology. H_2_S regulates vascular tone and oxidant defenses and exerts cytoprotective effects in the heart and circulation. Recent studies indicate that H_2_S modulates various components of metabolic syndrome, including obesity and glucose metabolism. This review will discuss studies exhibiting H_2_S -derived cardioprotective signaling in heart failure with reduced ejection fraction (HFrEF). We will also discuss the role of H_2_S in metabolic syndrome and heart failure with preserved ejection fraction (HFpEF).

## 1. H_2_S Therapy in Heart Failure with Reduced Ejection Fraction

Heart failure with reduced ejection fraction (HFrEF) is one of the leading causes of mortality in the United States, with a 5-year mortality rate of 75% [1]. Heart failure patients have reduced circulating H_2_S levels, and H_2_S levels progressively decline as heart failure symptoms worsen [2]. These diminished H_2_S levels have been recapitulated in several preclinical animal models of HFrEF [3]. In a mouse model of HFrEF, whereby mice underwent a transverse aortic constriction (TAC) procedure and subsequently developed HFrEF, there was > 60% reduction in circulating and cardiac hydrogen sulfide concentrations, highlighting the potential role of H_2_S as a cardioprotective signaling molecule [4]. Endogenously produced hydrogen sulfide exerts a myriad of cytoprotective actions in vivo, specifically in its role as an antioxidant through promoting Nrf2 and NRF-1 signaling [5,6,7] and augmenting nitric oxide-mediated signaling [8]. These signaling mechanisms provide a component of the antioxidant and anti-apoptotic effects that are observed with the administration of H_2_S donors in a broad range of disease models. In a cystathionine γ-lyase (CSE) knockout mouse model with attenuated circulating plasma H_2_S, cardiac injury was exacerbated following myocardial ischemia–reperfusion injury compared to wild-type mice. This deleterious phenotype was ameliorated following administration of a hydrogen sulfide donor. Interestingly, in an endothelial nitric oxide synthase (eNOS) phospho-mutant mouse, exogenous H_2_S failed to provide a protective effect in acute myocardial infarction, indicating the importance of eNOS-NO signaling in H_2_S-mediated protection [8]. These findings demonstrate hydrogen sulfide’s ability to activate or rescue eNOS functionality and may indicate a therapeutic niche for H_2_S in diseased states with high reactive oxygen species, such as heart failure, where eNOS is uncoupled into non-functioning monomers [8].

The mechanisms by which H2S activates eNOS involve promoting the recoupling of eNOS monomers, increasing the phosphorylation of eNOS at ser1177 while reducing the phosphorylation at thr495, and inhibiting endogenous eNOS inhibitors such as proline-rich tyrosine kinase 2 (PYK2) via s-sulfhydration [9]. Not only has H_2_S been shown to affect signaling trough sulfhydration, but it has also been investigated as a pan-inhibitor of phosphodiesterase, leading to a potential added benefit of increasing cGMP signaling in cardiovascular-diseased states [10]. This H_2_S mediated inhibition has been shown with decreases in both homodimers of PDE 5A and 5′-GMP content following incubation of rat vessels with NaHS or GYY4137 [11]. In addition, when human internal mammary arteries were exposed to NaHS, there was an observed increase in eNOS phosphorylation and subsequent decreased PDE5A level compared to that of arteries exposed to a Krebs control [12].

The tandem effects of H_2_S and NO, in addition to their metabolites and downstream signaling modulators, have been implicated in a range of cardioprotective effects [9,10,11,12,13]. Calvert et al. investigated the importance of CSE-mediated H_2_S generation in the setting of HFrEF. Myocardial CSE overexpression led to improved systolic function in a mouse model of ischemia-induced heart failure [6]. What remains unclear is whether the reduced H_2_S bioavailability observed in HFrEF is due to decreased enzymatic production through endogenous H_2_S-producing enzymes CSE, Cystathionine β-synthase (CBS) and 3-Mercaptopyruvate sulfurtransferase (3-MST) or due to increased metabolism of H_2_S, or both mechanisms.

Both polysulfides and persulfides have been shown to sulfhydrate cysteine residues of proteins in a much more potent manner compared to H_2_S [14]. Many novel polysulfide or persulfide compounds have been studied in the setting of various diseases [15,16,17,18,19]. However, due to the chemical nature of these compounds, they are also the source of H_2_S as a separate mechanism of action. Hence, it is very difficult to delineate their effects directly as a polysulfide or through its donation of H_2_S through extended chains of sulfur atoms. It is believed that these molecules themselves are in fact interchangeable in solution [20]. Nevertheless, several novel H_2_S-donating therapeutic compounds are known polysulfides [20,21,22,23,24].

SG-1002 is an alpha-sulfur polysulfide compound that also serves as a pro-H_2_S donor. In recent findings, wild-type mice and CSE-KO mice underwent TAC and then received either SG-1002 (P.O. in chow at 20 mg/kg/day) or vehicle. CSE-KO mice developed worsened cardiac remodeling and function compared to control mice. Interestingly, CSE-KO mice that received SG-1002 exhibited improved cardiac remodeling and function [6]. A subsequent phase 1 clinical trial was performed using SG-1002 for a 21-day period in healthy and heart failure subjects. The drug was well tolerated and SG-1002 effectively increased circulating H_2_S and nitrite levels [17]. To date, there are no other heart failure clinical trials that have been completed utilizing a H_2_S donor.

Sodium sulfide (Na_2_S) was one of the first H_2_S donors studied and has a very rapid release profile. Mice administered sodium sulfide 100 μg/kg/d through daily venous injections beginning 4 weeks after ischemia-induced heart failure had increased Nrf2 signaling along with preserved cardiac structure and function compared to vehicle control mice. Following the induction of ischemic heart failure in Nfr2-KO mice, sodium sulfide administration failed to alter cardiac function or structure compared to control. This indicates that H2S-mediated cardioprotection in ischemic heart failure is Nrf2-dependent [25]. It has also been reported that sodium sulfide leads to transient arterial pressure decreases when administered at 15 mg/kg i.v. [26]. The hemodynamic effects of H_2_S cannot be overlooked as a possible protective mechanism of action. Sodium sulfide has also been shown to upregulate Heme-oxygenase-1 in a volume overload mouse model of heart failure [27]. Reactive oxygen species in conjunction with cellular apoptosis are also decreased in HFrEF mice following Na_2_S administration [28]. These H_2_S-dependent antioxidant and anti-apoptotic effects have been reported in rodents in models of doxorubicin or anthracycline-induced heart failure [29].

Diallyl trisulfide (DATS) is a garlic-derived polysulfide that has a H_2_S-releasing profile that is more sustained than traditional H_2_S donors such as sodium sulfide. In a 12-week study of TAC-induced heart failure, DATS therapy (200 µg/day) led to improved LV ejection fraction compared to vehicle. Mice receiving DATS also had increased cardiac microvessel density, increased VEGF signaling, and attenuated myocardial fibrosis [16]. In addition, under certain conditions, DATS may react with its existing cysteine sulfhydyl residues forming trisulfide metabolites and elicit cytoprotective effects independent of H2S formation [30].

Li et al. studied the cardioprotective effects of a novel H_2_S donor, JK-1, in a TAC-induced murine heart failure model. JK-1 releases H_2_S in a pH-dependent manner via a hydrolysis reaction and this release profile is ideally suited to pathological CV disease states such as heart failure. Delayed treatment of JK-1 following the onset of heart failure led to reduced left ventricular dilation and improved LV ejection fraction compared to a control group [31]. This study was also the first described evidence that H_2_S attenuates the activation of the renin–angiotensin–aldosterone system (RAAS) in the setting of heart failure. Downregulation of RAAS ultimately led to reduced renal fibrosis and improved renal function in the JK-1-treated group.

Wu et al. first described the novel compound, ZYZ-803, which activates both CSE and eNOS, leading to increased H_2_S and nitric oxide production simultaneously. ZYZ-803 has been tested in a mouse heart failure model induced by isoprenaline. Following administration of ZYZ-803, the mice had improved cardiac structural changes and increased LV ejection fraction compared to that of the vehicle group [32]. The drug appeared to effectively activate CSE and eNOS as plasma H_2_S and nitric oxide levels were increased at the study endpoint. Cardioprotective effects of ZYZ-803 were diminished with simultaneous administration of the eNOS inhibitor, L-NAME, and the CSE inhibitor, DL-propargylglycine (PAG). These results reveal that concurrent activation of these enzymes produces a greater effect than either one alone.

The slow-releasing H_2_S donor, GYY4137, has been shown to preserve left ventricular ejection fraction 7-days post-MI compared to that of control. Interestingly, despite improved cardiac function, these animals also had increased natriuretic peptide levels compared to control. The mechanism of H_2_S-mediated augmentation of natriuretic peptides remains unknown. In this same model, GYY4137 therapy led to increased NO-cGMP signaling post-MI [33]. In isolated cardiomyocytes infected with coxsackie virus, GYY4137 was also shown to produce anti-inflammatory actions through decreased NFKB and MAPK signaling [34].

**Figure 1:**
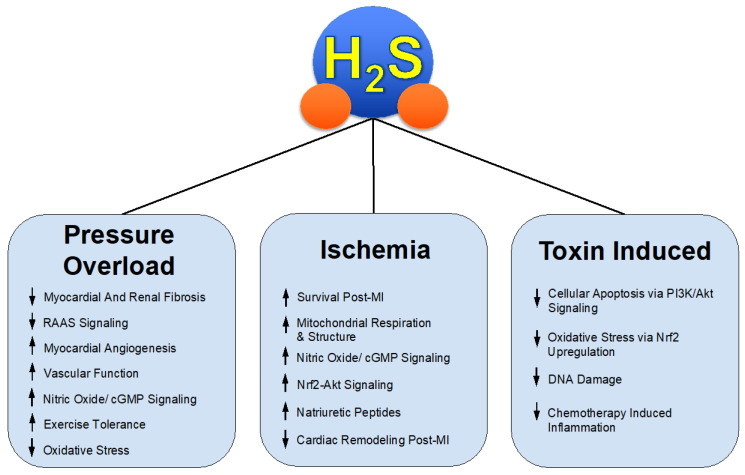
H_2_S Mediated Protection in Heart Failure.

Through both the investigation and success of these various H_2_S donors in relevant pre-clinical models such as HFrEF, we gain insight into the mechanisms of protective effects in pathological states seen in Figure 1. H_2_S therapy might be applied to similar states of not only pathological cardiac remodeling but a myriad of disease states involving, but not limited to, systemic inflammation, fibrosis, vascular diseases such as hypertension and peripheral artery disease [35,36,37,38], and overactive RAAS signaling.

## 2. H_2_S Therapy in the Setting of Metabolic Syndrome

**Figure 2:**
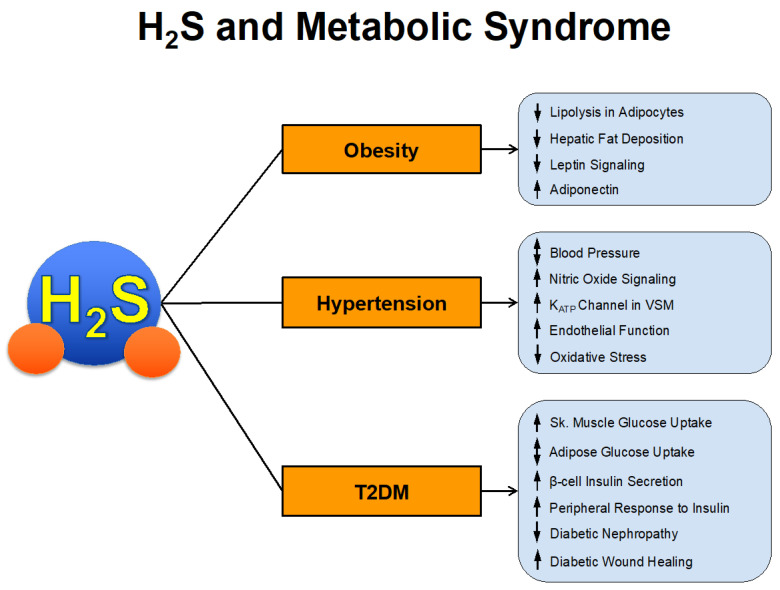
H2S and Metabolic Syndrome.

The prevalence of metabolic syndrome has increased at an alarming rate over the past several decades. It is estimated that approximately 1/3 of US adults, 12–37% of the Asian population, and 12–26% of the European population have metabolic syndrome [39,40]. These numbers equate to > 1 billion people worldwide. Those with metabolic syndrome have increased risk of cardiovascular morbidity and mortality. In fact, estimates suggest that 6–7% of all-cause mortality and 12–17% of cardiovascular disease can be attributed to metabolic syndrome [41]. In a survey of US adults, metabolic syndrome had an increased hazard ratio for coronary heart disease mortality of 2.02 and all-cause cardiovascular disease mortality of 1.82 [42]. We will now summarize the studies that have investigated the direct and indirect actions of H_2_S in the various components of metabolic syndrome summarized in Figure 2.

### 2.1. H_2_S and Obesity

Obesity is a leading contributor to metabolic disease by numerous mechanisms. Obesity results in the release of increased nonesterified fatty acids (NEFAs), which, when acting on skeletal muscle, can promote insulin resistance and the development of fatty liver disease. Adipose tissue also synthesizes and secretes inflammatory cytokines, including TNF-alpha and IL-6, which are correlated with increased cardiovascular risk. Although adipose cells produce leptin, which is a hormone that helps to regulate the energy balance by inhibiting hunger and fat storage metabolism, there is leptin resistance in obese patients. Conversely, obese persons generally have lower levels of adiponectin, which is a hormone produced primarily in adipose tissue, with anti-inflammatory and anti-atherogenic properties.

It is unclear to what degree H_2_S regulates lipolysis or the development and pathology of obesity and metabolic syndrome. Some studies have suggested that H_2_S inhibits lipolysis, while others suggest that upregulation of H_2_S signaling stimulates adipose tissue lipolysis. Geng et al. reported that an H_2_S synthesis inhibitor, DL-propargylglycine (PAG), increased isoproterenol-stimulated lipolysis in rat adipocytes, while the H_2_S donor, GYY4137, reduced adipose tissue lipolysis [43]. In another study, sodium sulfide (Na_2_S) was administered via microdialysis probe into subcutaneous adipose tissue and lipolysis was estimated by measuring glycerol levels [44]. In a dose-dependent manner, Na_2_S led to increased glycerol production, which was accompanied by cAMP release. This release was abolished by the protein kinase A (PKA) inhibitor, KT5720, indicating that H_2_S-induced lipolysis is PKA-dependent. H_2_S-mediated glycerol release was greater in rats that were fed a high-fat diet. They also found that an inhibitor of H_2_S release (PAG) resulted in decreased glycerol production in obese rats.

The three H_2_S-generating enzymes, CSE, CBS, and 3-MST, are all produced in the liver and contribute to hepatic H_2_S production and hepatic physiology. Malfunction of hepatic H_2_S metabolism is involved in the pathogenesis of many liver diseases and H_2_S regulates lipid metabolism [45]. Studies have suggested that exogenous H_2_S improves fatty liver disease by improving lipid metabolism. In high-fat-diet-induced obese mice, daily injection of the H_2_S donor, NaHS, for 4 weeks resulted in improved hepatic cellular structure, decreased liver weight and Oil-red-O staining, and decreased triglycerides and total cholesterol [46]. The endogenous role of H_2_S signaling appears to center around the interplay of the H_2_S-producing enzymes. Meng et al. report that hepatic expression of 3-MST is upregulated in patients with non-alcoholic fatty liver disease as well as in mice fed a high-fat diet [47]. However, this increase in 3-MST led to a paradoxical decrease in H_2_S production because 3-MST directly interacted with and negatively regulated CSE in the liver. Furthermore, inhibition of 3-MST significantly enhanced, rather than decreased, H_2_S production and reduced FFA-induced fat accumulation in L02 cells.

Adipose tissue is also an endocrine organ and the critical signaling hormones produced by adipose are leptin and adiponectin. Leptin resistance characterized by elevated circulating leptin levels is a hallmark of obesity. The effects of exogenous H_2_S in leptin signaling have been previously investigated. Wu et al. reported that pre-treatment of human umbilical vein endothelial cells with the H_2_S donor, sodium hydrosulfide, prior to glucose challenge resulted in decreased leptin as well as decreased leptin receptor expression [48]. Moreover, Zhuang et al. report that exogenous NaHS prevents high-glucose-induced injury by inhibiting the leptin-p38 MAPK signaling pathway in H9c2 cells. This group also reports that H_2_S attenuates leptin and leptin receptor expression [49]. On the other hand, circulating levels of adiponectin are negatively correlated with obesity and have been shown to be protective in cardiovascular disease [50]. Interestingly, there appears to be a positive correlation between circulating H_2_S and adiponectin levels in humans [24]. High glucose concentration has also been shown to decrease the expression of the H_2_S-producing enzyme, CSE, and lead to the downregulation of adiponectin [51]. Conversely, overexpression of CSE or exogenous treatment with NaHS resulted in increased adiponectin in adipocytes, indicating that H_2_S and adiponectin are not merely correlating bystanders but that H_2_S directly causes increased adiponectin production.

### 2.2. H_2_S and Type 2 Diabetes Mellitus

Type 2 diabetes mellitus (T2DM) carries a significant and increasing disease burden worldwide. Complications can be broadly categorized into microvascular and macrovascular complications. Microvascular complications, largely due to the accumulation of advanced glycosylation end-products and vascular injury, include retinopathy, nephropathy, neuropathy, and poor wound healing. Macrovascular complications of diabetes mellitus include increased risk of stroke, heart disease, and peripheral vascular disease. Interestingly, alterations in H_2_S signaling seem to play a role in glucose handling and the pathogenesis of T2DM.

Multiple groups have reported that patients with T2DM have lower circulating H_2_S levels than their age-matched non-diabetic counterparts [24,52]. Interestingly, H_2_S appears to decrease pancreatic beta-cell secretion of insulin but increase peripheral response to insulin-mediated glucose uptake peripherally [53]. In the liver, H_2_S likely mobilizes glucose stores by increasing gluconeogenesis and glycogenolysis. The interplay between H_2_S and NO in glucose regulation is somewhat unclear since studies report some discordant actions of NO compared to H_2_S. Similarly to H_2_S, NO promotes peripheral glucose uptake by skeletal muscle; however, its actions in the pancreas and liver differ. NO increases pancreatic insulin secretion by beta-cells. In the liver, NO inhibits glucose mobilization and promotes glucose storage by decreasing gluconeogenesis and glycolysis [54]. As we discussed earlier, H_2_S promotes NO signaling; thus, studies identifying which molecule plays a predominant role in glucose metabolism are warranted.

Several pre−clinical studies provide evidence that H_2_S attenuates many of the complications of T2DM. Wound healing is impaired in diabetic patients, largely due to impaired microvascular function and localized inflammation. Wang et al. found that localized daily treatment with bisulfide ointment for 21 days improved wound healing in diabetic rats [55]. They found that in these animals, H_2_S promoted angiogenic signaling while reducing markers of oxidative stress and inflammation. Diabetic nephropathy, and ultimately chronic kidney disease, develops because glycosylation of the vascular basement membrane leads to hyaline arteriolosclerosis. The efferent arteriole is preferentially affected, which leads to increased glomerular filtration pressure. Hyperfiltration leads to microalbuminuria and eventual progression to nephrotic syndrome. In a streptozotocin-induced diabetic rat model, Zhou et al. report that daily injections of NaHS for 12 weeks led to improved renal function, attenuated glomerular basement membrane thickening, and blunted interstitial fibrosis and mesangial matrix deposition [56]. Using the same rat model of streptozotocin-induced diabetes, Sun et al. demonstrated that the sulfhydration of SIRT1 by Na_2_S_4,_ a polysulfide compound, leads to ameliorated diabetic nephropathy [19]. As in many of the studies examining H_2_S in various models of metabolic syndrome, there are no identifiable clinical trials that are examining or have examined the effects of an H_2_S donor in patients with T2DM. We believe that hydrogen sulfide, with its diverse mechanisms of action in rigorously performed in vitro and in vivo animal models, is an ideal candidate for the treatment of complications from T2DM.

### 2.3. H_2_S and Blood Pressure Regulation

H_2_S regulates vascular function and hemodynamics, but the precise role of H_2_S in vascular function remains unclear. Some studies report vasodilatory actions of H_2_S while others report vasoconstriction. It is possible that these discrepancies can be explained by dose-dependent effects, which vascular beds are under investigation, or the oxygen tension within that vascular bed. CSE KO mice, which are consequently deficient in H_2_S, are significantly hypertensive and have impaired endothelium-mediated vasorelaxation [57]. Exogenous H_2_S has been shown to act as a vasodilator at lower oxygen pressures (30 mmHg) and as a vasoconstrictor at elevated partial pressure of oxygen (150 mmHg) [58]. This discordance may suggest that H_2_S would have more dilatory effects on the venous side of the circulatory system with lower oxygen partial pressures. H_2_S appears to also play a role in the pulmonary vasculature, particularly in the setting of hypoxic pulmonary hypertension. Chunyu et al. reported that CSE expression in the lungs and plasma H_2_S levels are both decreased in hypoxia-induced pulmonary hypertension and suggest that exogenous H_2_S could oppose this rise in pulmonary arterial pressures [59].

There does not appear to be a single mechanism by which H_2_S modulates vascular tone; however, several have been proposed. It is likely that multiple signal transduction pathways are activated in the endothelium and vascular smooth muscle cells. Studies by Naik et al. suggest that H_2_S activates endothelial TRPV4 channels which allow for Ca^2+^ influx and subsequent vasodilation. Another mechanism of H_2_S-regulated blood pressure reduction may be its interplay with the potent vasodilator, nitric oxide (NO). Studies have shown that exogenous hydrogen sulfide activates eNOS and increases nitric oxide signaling [16]. Additionally, suppression of hydrogen sulfide production via CSE gene suppression leads to inhibited eNOS activity and diminished NO signaling. Others suggest that H_2_S-induced vasorelaxation is dependent on the activation of ATP-sensitive K+ channels in vascular smooth muscle [60,61]. In these studies, concentration-dependent H2S-induced vasodilation was inhibited by the K_ATP_ channel blocker glibenclamide [62]. However, another mechanism of H_2_S-mediated vascular regulation is its action as a powerful antioxidant. Oxidative stress causes endothelial dysfunction and has detrimental effects on vascular function and tone. Several models of elevated oxidative stress, including neuronal ischemia–reperfusion injury, myocardial ischemia–reperfusion injury, heart failure, and limb ischemia, report that H_2_S donors attenuate local and systemic oxidative stress and improve cellular health [6,14,63]. H_2_S scavenges O^2−^ and reduces vascular NADPH oxidase-derived superoxide anion production [64]. H_2_S also inhibits H_2_O_2_-mediated mitochondrial dysfunction by preserving the activity of superoxide dismutase, catalase, glutathione peroxidase, and glutathione-S-transferase while promoting mitochondrial biogenesis via sulfhydrating PP2A and activating AMPK [65,66].

An important question is whether chronic H_2_S therapy could have consistent and sustained actions on blood pressure control. Many of the studies reported in the literature describe the acute vasodilatory effects of a H_2_S donor bolus. Numerous studies also discuss the development of hypertension in models of H_2_S deficiency. However, we have yet to find conclusive studies reporting that H_2_S can safely and effectively reduce blood pressure in the setting of chronic systemic hypertension. A likely reason for this is the release profile of most of the H_2_S donors. Most of these compounds release H_2_S in the order of seconds to minutes, which would unlikely be clinically therapeutic in a chronic disease state where effective treatment requires blood pressure reduction throughout the entire day [2]. However, as discussed, a few of the proposed blood-pressure-lowering effects, specifically the role of H_2_S as an antioxidant and an eNOS activator, may lead to more sustained anti-hypertensive effects than the abrupt H_2_S release profile of many of the developed compounds. Further studies in models of chronic hypertension are required to answer these questions.

## 3. The Potential of H_2_S Therapy in HFpEF

Heart failure with preserved ejection fraction (HFpEF) is a complex heterogeneous multi-organ disease that has been at the forefront of heart failure research over the past decade. HFpEF is the most common of all heart failure (HF) diagnoses, expected to amass > 60% of all clinical HF cases by 2030 [67]. In contrast to HFrEF, the list of disappointing HFpEF clinical trials is expanding, with no FDA-approved pharmacotherapies showing significant decreases in patient hospitalization or mortality rates. These failures contribute to HFpEF being widely regarded as the largest unmet clinical need in cardiovascular medicine [1,68].

HFpEF therapy development is further complicated by its heterogeneous phenotype and non-unified diagnostic criteria [1,69]. Until recently, HFpEF was broadly defined as a patient experiencing symptoms of HF while sustaining normal left ventricular ejection fraction (> 50%) [70]. Given the generality of the HFpEF diagnosis, the current treatment regimen lacks precision and is largely limited to symptom management targeting the accompanying comorbidities.

In-depth investigation into HFpEF clinical presentations has led to the identification and stratification in three distinct phenogroups that include: (1) elderly patients with moderate diastolic dysfunction, hypertension, and relatively preserved brain natriuretic peptide (BNP), (2) obese, diabetic patients with significantly impaired left ventricular relaxation, and (3) older patients with chronic kidney disease, pulmonary hypertension, and right ventricular dysfunction. Stratification into these phenotypes revealed correlative comorbidities and mortality rate among groups [71,72]. The identification of multiple phenogroups within the HFpEF population has raised the possibility that a single mechanistic treatment approach likely will not yield optimal outcomes in this heterogeneous syndrome.

Phenogroup 2 HFpEF patients suffer from severe cardiometabolic disease and have the highest incidence of obesity, diabetes mellitus, and obstructive sleep apnea. Moreover, > 50% of this HFpEF subgroup contains patients with dyslipidemia, type 2 diabetes mellitus, hypertension, and/or obesity [71]. The combination of these comorbidities leads to metabolic syndrome, an energetically dysregulated state known to cause deleterious cardiometabolic effects [41,73]. As described previously, H_2_S has been shown to positively modulate obesity, dyslipidemia, glucose control, and insulin resistance. Therefore, H_2_S therapy may improve outcomes in phenogroup 2 HFpEF patients by addressing the deleterious accompanying comorbidities. It is also possible that H_2_S may have direct myocardial cardioprotective actions on the heart, as it does in HFrEF. Specifically, H_2_S has been shown to decrease cardiac fibrosis, which is critically important in treating a disease state whose hallmark is a non-compliant ventricle [3]. Given that H_2_S has been previously researched as a treatment strategy for these specific HFpEF-inducing comorbidities, as well as its involvement in HFrEF progression, we identify this specific phenotype as the most interesting candidate to investigate the use of H_2_S as an effective therapeutic approach [74].

Cardiometabolic HFpEF poses an interesting challenge in HFpEF research in that systemic metabolic dysregulation, vascular dysfunction, inflammation, and oxidative stress predominate this disease. The accumulation of these systemic insults, though initially compensated by increasing the work of the heart, eventually results in overwhelming systemic stress, leading to significant left ventricular diastolic dysfunction. The predominance of metabolic and inflammatory disturbances in cardiometabolic-HFpEF makes it a most interesting target for H_2_S therapy, especially regarding the potential effect that H_2_S will have on attenuating the pro-inflammatory metabolic state.

**Figure 3:**
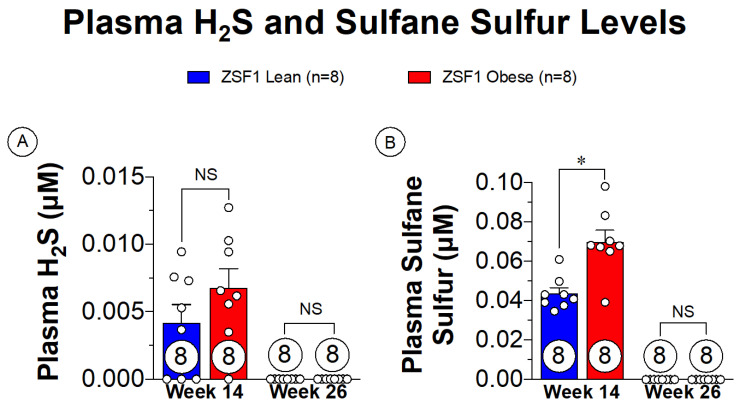
Plasma free H_2_S and sulfane sulfure levels in ZSF1 obese rats prior at multiple time points in disease progression. (**A**) Plasma H_2_S levels at 14 weeks of age (prior to HFpEF onset) and at 26 weeks of age (late-stage HFpEF). (**B**) Plasma sulfane sulfur levels at 14 and 26 weeks of age. NS: Not Significant, * *p* < 0.05.

Small animal modeling of cardiometabolic-HFpEF has been thoroughly described with the ZSF1 obese rat [75,76]. The ZSF1 obese rat presents with severe HFpEF signs and symptoms as early as 20 weeks of age [76]. The rapid development of the disease also makes the ZSF1 obese rats ideal for assessing the progression of HFpEF from asymptomatic to severe presentations. Plasma sulfane sulfur is a metabolic breakdown product of H_2_S that is a direct indication of H_2_S levels, allowing for a less volatile marker of H_2_S in the plasma [8]. Sulfane sulfur levels both prior to onset of HFpEF symptoms and in late-stage HFpEF are shown in Figure 3. With the presence of the metabolic disturbances at the 14-week timepoint, there are extremely low levels of plasma sulfane sulfur present. Of significant importance, however, is that the progression of HFpEF symptoms depleted the remaining sulfane sulfur levels. These observations highlight a novel finding in the setting of cardiometabolic-HFpEF, suggesting that therapeutic supplementation with H_2_S donors could potentially benefit both metabolic and cardiovascular components of this disease.

## 4. Conclusions

Attempts to treat the systemic pathology associated with HFpEF using therapeutics previously approved for HFrEF have failed to provide positive results despite their clear-cut benefits in HFrEF. This is likely due to the heterogeneity of the HFpEF patient population and the highly complex and diverse pathology of this disease. Stratification of HFpEF patient populations and the use of combination therapies in future clinical studies may reveal a viable therapeutic solution. We propose that the previously described actions of H_2_S, including its global involvement with metabolic syndrome, RAAS, sympathetic output, blood pressure, and cytoprotectant effects, make it a worthy candidate for future investigation as a treatment for cardiometabolic-HFpEF and perhaps additional HFpEF phenotypes.

Through previous animal modeling in HFrEF, H_2_S has been shown to provide cytoprotective effects through NRF2- and eNOS-dependent pathways, alleviating systemic oxidative stress and improving vascular function found in pathological inflammatory states. In investigating models of obesity and metabolic syndrome, hydrogen sulfide has been shown to not only beneficially impact circulating lipids but improve glucose signaling in a diabetic state. With regard to hypertension, H_2_S has been shown to improve endothelial function and nitric oxide signaling, with mixed reports regarding overall blood pressure regulation. In the HFpEF ZSF1 rat model (Figure 3), we have now shown that circulating plasma sulfane sulfur levels are severely diminished during the progression of this disease, providing some new evidence that loss of H_2_S may be involved in the pathogenesis of HFpEF. Additional studies are required to determine if reductions in H_2_S in fact contribute to HFpEF pathology.

While this review emphasizes the therapeutic role of H_2_S supplementation in heart failure and metabolic syndrome, it must also be mentioned the potentially toxic role of H_2_S in excess. H_2_S, along with other signaling molecules, must remain in a homeostatic concentration in the body, stressing the importance and potential danger of supplementing H_2_S with supraphysiologic levels. It is also worth noting that the potential role of contamination of NaHS in solutions, although not investigated thoroughly, is an ongoing issue in the H_2_S field [77].

Although H_2_S has been shown to be beneficial in these models of HFrEF and metabolic syndrome, further studies are required to investigate its true role in the setting of HFpEF pathology. It may be that several therapeutics are necessary to combat the heterogeneity of this disease, where H_2_S may contribute in a supportive role.
